# Supplementary material for: Functional architecture of executive control and associated event-related potentials in macaques
Source: Nat Commun. 2022 Oct 21;13:6270. doi: 10.1038/s41467-022-33942-1 (PMC9586948; doi:10.1038/s41467-022-33942-1)
Supplement: Supplementary file 1 — Supplementary Information [file 41467_2022_33942_MOESM1_ESM.pdf]

Supplementary Information

Functional architecture of executive control and associated event-related potentials in macaques

Amirsaman Sajad[1*], Steven P. Errington[1*], & Jeffrey D. Schall[1,2]

[1] Department of Psychology, Vanderbilt Vision Research Center, Center for Integrative & Cognitive Neuroscience, Vanderbilt University, Nashville, TN

[2] Centre for Vision Research, Vision Science to Application, Department of Biology, York University, Toronto, ON

[*] Authors contributed equally to this work.

Supplementary Figure 1 | SEF laminar structure and neuron classification.

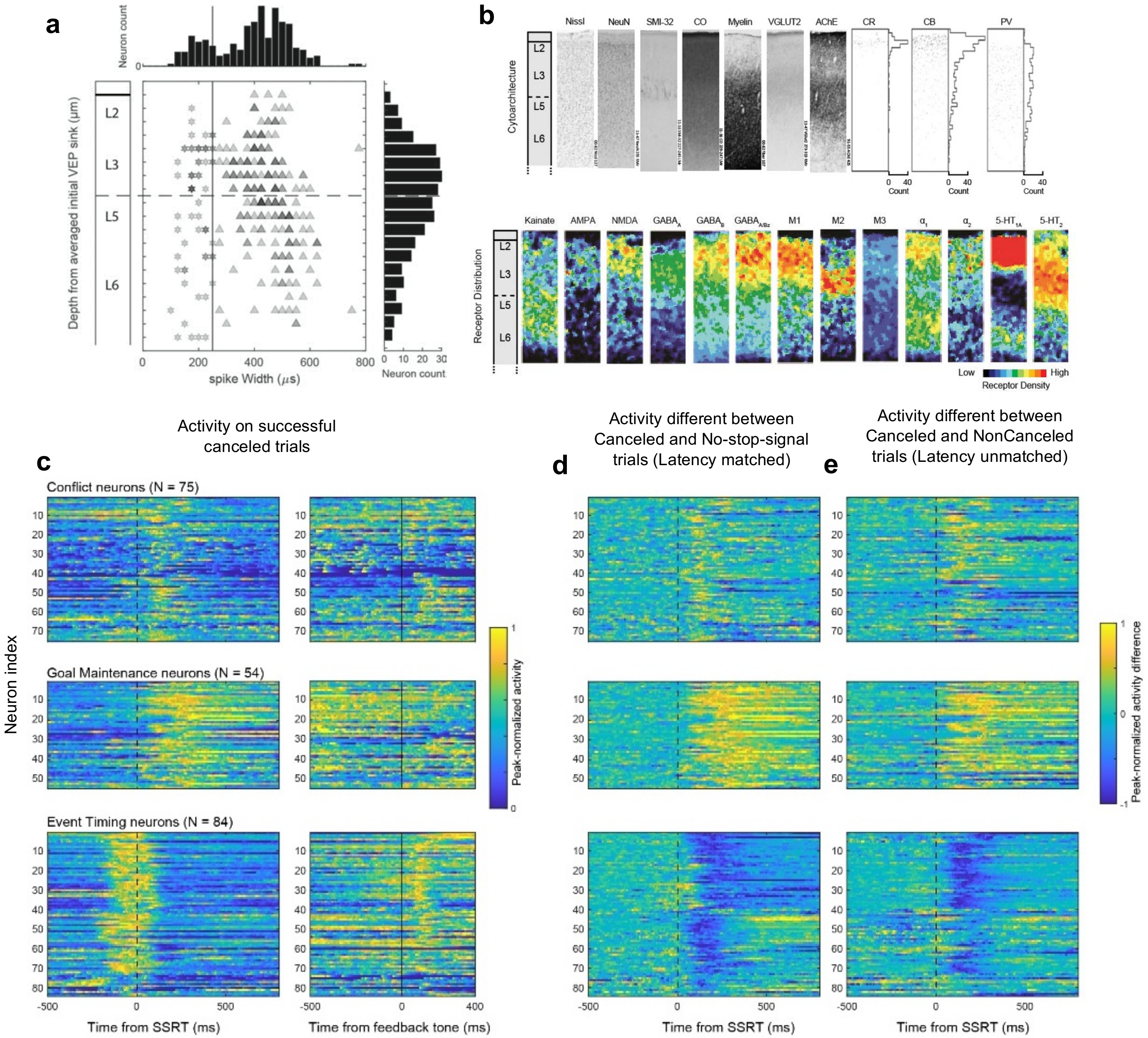

a, Distributions of depths of units and spike widths sampled. Horizontal histogram shows the spike widths of all neurons sampled (n = 575), which exhibited bimodal values. The vertical line marks the 250 µs separation criterion used. Scatterplot shows variation of spike width across depth for neurons sampled in perpendicular penetrations (n = 293). Narrow spiking neurons are indicated by stars and broad spiking neurons by triangles. The number of neurons at each time-depth indicated by gray scale (darker indicating higher count). The width of the spikes narrower than 250 µs does not vary with depth, and the incidence of encountering narrow spikes parallels the density of parvalbumin (PV) neurons [1]. The width of spikes wider than 250 µs increases from L3 to L6, which parallels the size of pyramidal neurons. Also, the

incidence of isolated neurons decreases with depth, which parallels the density of pyramidal neurons. Vertical histogram shows the depths of all neurons sampled during the saccade countermanding task from the sessions with penetrations oriented perpendicular to the cortical layers. The color scale used for the time-depth plots in Fig. 2, 3, 5, and 5 indicates the number of neurons of each class sampled relative to the entire sampling distribution. Adapted from [2].

b, Laminar structure of SEF. Top row, from left to right are shown sections through SEF stained for Nissl, NeuN, nonphosphorylated neurofilament H (SMI-32), cytochrome oxidase (CO), Gallyas myelin, vesicular glutamate transporter 2 (VGLUT2), acetylcholinesterase (AChE), calretinin (CR), calbindin (CB), and PV. Counts of CR, CB, and PV-stained neurons are adapted from [1]. In SEF, L1 is ~200 µm thick with some CR and CB but no PV neurons, and weak staining for CO, myelin, and VGLUT2. L2 is ~300 µm thick with dense stellate and few pyramidal neurons, the highest density of CR and CB neurons, but no SMI-32, stronger CO staining, fascicular myelin fibers, slightly stronger VGLUT2, and modest AChE. L3 is ~700 µm thick with a superficial sublayer with smaller pyramidal neurons and weak SMI-32 staining, very few CR, and modest densties of CB and PV neurons, stronger CO staining and denser myelin, VGLUT2, and AChE. A deeper sublayer is characterized by larger pyramidal neurons, pronounced SMI-32 staining, vanishingly few CR, less dense CB and modestly dense PV neurons, weaker CO, denser myelin and VGLUT2, and denser AChE. No granular L4 is evident in SEF. L5 is ~300 µm thick with large pyramidal neurons but inconsistent SMI-32 staining, no CR and fewer CB but modest density of PV neurons, lighter CO, denser myelin, lighter VGLUT2, and diminishing AChE staining. L6 is ~700 µm thick with smaller pyramidal neurons, light SMI-32, no CR, vanishingly few CB and low density of PV neurons, with lighter CO, still denser myelin, lighter VGLUT2 and sparse AChE staining.

Lower row, sections processed for receptor autoradiography and color coded to visualize the laminar densities of various receptors adapted from [3]**.** The color scale maps to densities in fmol/mg protein with (minimum, maximum) levels as indicated for the following receptors: kainate (20, 1200), AMPA (20, 750), NMDA (200, 2500), $GABA_A$ (100, 3200), $GABA_B$ (150, 3500), $GABA_{A/Bz}$ (200, 3500), muscarinic M_1 (100, 1200), muscarinic M_2 (10, 350), muscarinic M_3 (100, 1200), adrenergic α_1 (50, 700), adrenergic α_2 (20, 800), serotinergic 5-HT_{1A} (20, 700), and serotinergic 5-HT_2 (100, 500). Receptor densities reveal pronounced differences between L2/3 and L5/6 and other differences distinguishing L2 from L3 and L5 from L6. The variation in laminar structure can guide the investigation and interpretation of functional architecture of SEF.

c-e, Heat-maps representing normalized spike-density function for all neurons reported in this paper. These neuron classes emerged from our neuron classification pipeline (**Fig. 1c**). In a sample of 575 neurons, we took the following steps to identify distinct types of neurons. First, we identified 271 with significant changes in spiking rate following presentation of the stop-signal until 200 ms after stop-signal reaction time (SSRT) on successfully canceled trials. Second, the spike density function on canceled and latency-matched no-stop-signal trials in intervals 0–100 ms before and 0–200 ms after SSRT were submitted to an unsupervised consensus clustering pipeline [4]. 5 clusters were identified. Neurons were distinguished by a relative increase or decrease in discharge rate on canceled compared to latency-matched no-stop-signal trials. Cluster 1 included 146 neurons; cluster 2, 65 neurons; cluster 3, 28 neurons; and cluster 4, 16 neurons, and cluster 5, 16 neurons. Clusters 1 and 2 formed the majority of neurons and were analyzed further. The clustering results were verified by manual curation, resulting in some cluster 3, 4, and 5 neurons being included in clusters 1 and 2 and removal of neurons with poor signal quality. Therefore, this clustering procedure justified the consideration of two general types of modulation: facilitation (i.e., relatively higher activity on canceled trials) and suppression (i.e., relatively lower activity on canceled trials). Within each cluster we observed heterogeneity in the latency, duration, and magnitude of modulation after SSRT and around the time of feedback tone. Tone modulation was indexed by the contrast (difference divided by sum) between spike counts 200 ms before and 200 ms after the feedback tone. Each neuron was located in a 3D space based on these values. K-means clustering of the normalized values distinguished two clusters among facilitated neurons primarily based on modulation duration. Arriving at k = 2 clusters was justified by the Elbow method (analysis of with-in sum of distance to center) and the Silhouette score (inset panel, **Fig 1c**). K-means clustering did not distinguish among the suppressed neurons (not shown). Thus, this rigorous classification approach resulted in three neuron classes based only on the pattern of modulation around the time of SSRT with no presumptions about their functional role. The distinct patterns of modulation were reinforced by qualitative examination of clear differences among population spike density functions (**Fig. 3, 4, 5**). The functional roles and further distinctions based on different patterns of modulation around the time of tone are described in the main text.

c, Heat-maps representing normalized spike-density function on successful canceled trials for all neurons reported in this paper. The spike-density function for each neuron is normalized to its peak activity ±300 ms around SSRT. The left panel plots activity aligned on stop-signal reaction time (SSRT) (dashed black line). The right panel plots activity aligned on the feedback tone (solid line). Each row corresponds to activity of one neuron with higher discharge rates in hotter, and lower discharge rates in cooler colors. The time course of activation that distinguished conflict, event-timing, and goal maintenance neurons is evident in the three pairs of panels. Similarities and differences between these SEF neurons and

DA neurons in SNpc and neurons in striatum sampled during saccade countermanding is afforded by comparison with Fig. 2 and 5 of [5].

d, Heat-maps representing the difference in spike-density function on successful canceled trials relative to latency-matched no-stop-signal trials, aligned on SSRT, selected for the early stop-signal delay (SSD). The difference function for each neuron is normalized to the maximum deviation. Hot colors represent facilitation and cold colors represent suppression on canceled relative to no-stop-signal trials. The patterns of activation that distinguish the three major categories of neurons are more evident across the three panels. Similarities and differences between these SEF neurons and DA neurons in SNpc and neurons in striatum sampled during saccade countermanding is afforded by comparison with Fig. 2 and 5 of [5].

e, Heat-maps representing the difference in spike-density function on successful canceled trials relative to noncanceled trials (despite the inherent difference in underlying RT), aligned on SSRT, selected for an intermediate SSD with enough trials of both types. The patterns of activation that distinguish the three major categories of neurons are more evident across the three panels. Similarities and differences between these SEF neurons and DA neurons in SNpc and neurons in striatum sampled during saccade countermanding is afforded by comparison with Fig. 2 and 5 of [5].

Supplementary Figure 2 | Rationale for analysis of neural activity.

a

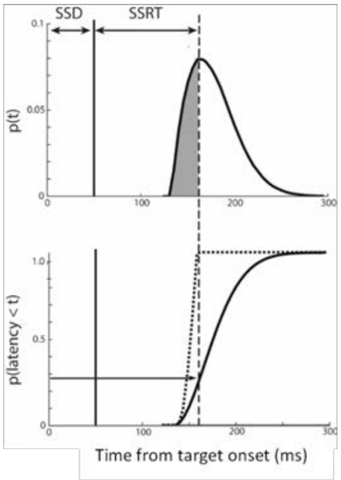

b

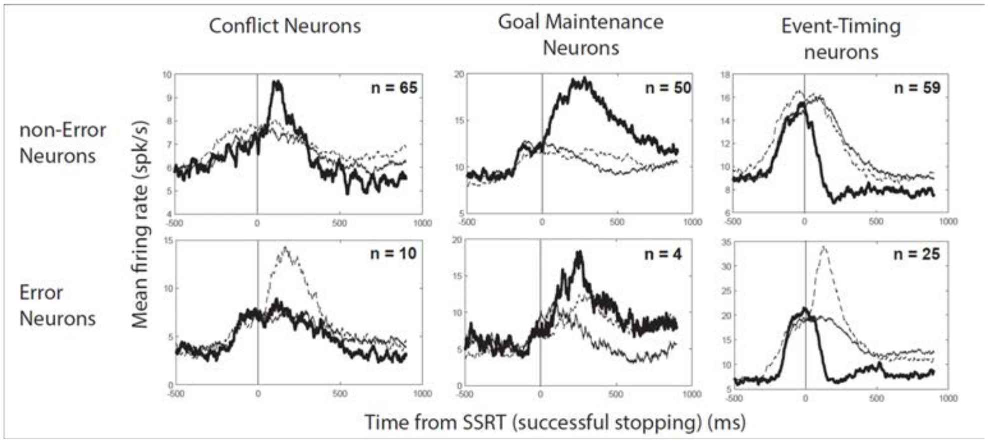

a, The primary analysis was based on the principles of the Logan race model, which have been used extensively [6-8]. Diagram of density (top) and cumulative (bottom) distributions of RT on trials with no stop-signal portrays the application of the race model for a given SSD. Because performance is the outcome of a race, at a given SSD, the finish time of the STOP process (SSRT) is the RT of trials with no stop-signal at which the fraction of the all trials equals p(NC | SSD) (shaded in density function and dotted in cumulative distribution). The race model entails that the no-stop-signal trials with RT > SSD + SSRT are so slow that they would have been canceled if the stop-signal had appeared. Therefore, we compared neural activity on canceled trials when response inhibition happened with activity on no-stop-signal trials when no response inhibition happened but with RT long enough that if the stop-signal had been presented, the saccade would have been canceled. By latency-matching canceled trials to no-stop-signal trials with RT > SSD + SSRT, the dynamics of the neural processes governing saccade initiation are equated. Likewise, noncanceled trials can be latency matched to no-stop-signal trials with RT < SSD + SSRT.

b, Activity on no-stop-signal (thin solid line), canceled (thick solid line), and error noncanceled (thin dashed) trials for neurons classes with post-SSRT modulation. Modulation related to successful stopping was determined based on the difference in activity between canceled and latency-matched no-stop-signal trials. Noncanceled trials were not used for analyzing modulation related to response inhibition for two reasons. First, failures of response inhibition in this task happen when RT is too short, which is the opposite of how successful inhibition occurs. Second, noncancelled trials elicit additional processes related to error detection. To illustrate, we have divided each neuron class into those without (top) and with (bottom) error-related modulation. The minority of Conflict (10/75), Goal Maintenance (4/54) and Event timing neurons (25/84) produced an error signal. Also, post-saccadic modulation in noncanceled and no-stop-signal trials is similar for neurons without but not with error-related modulation.

Supplementary Figure 3 | Time parameters and their relationships used to test neural modulation.

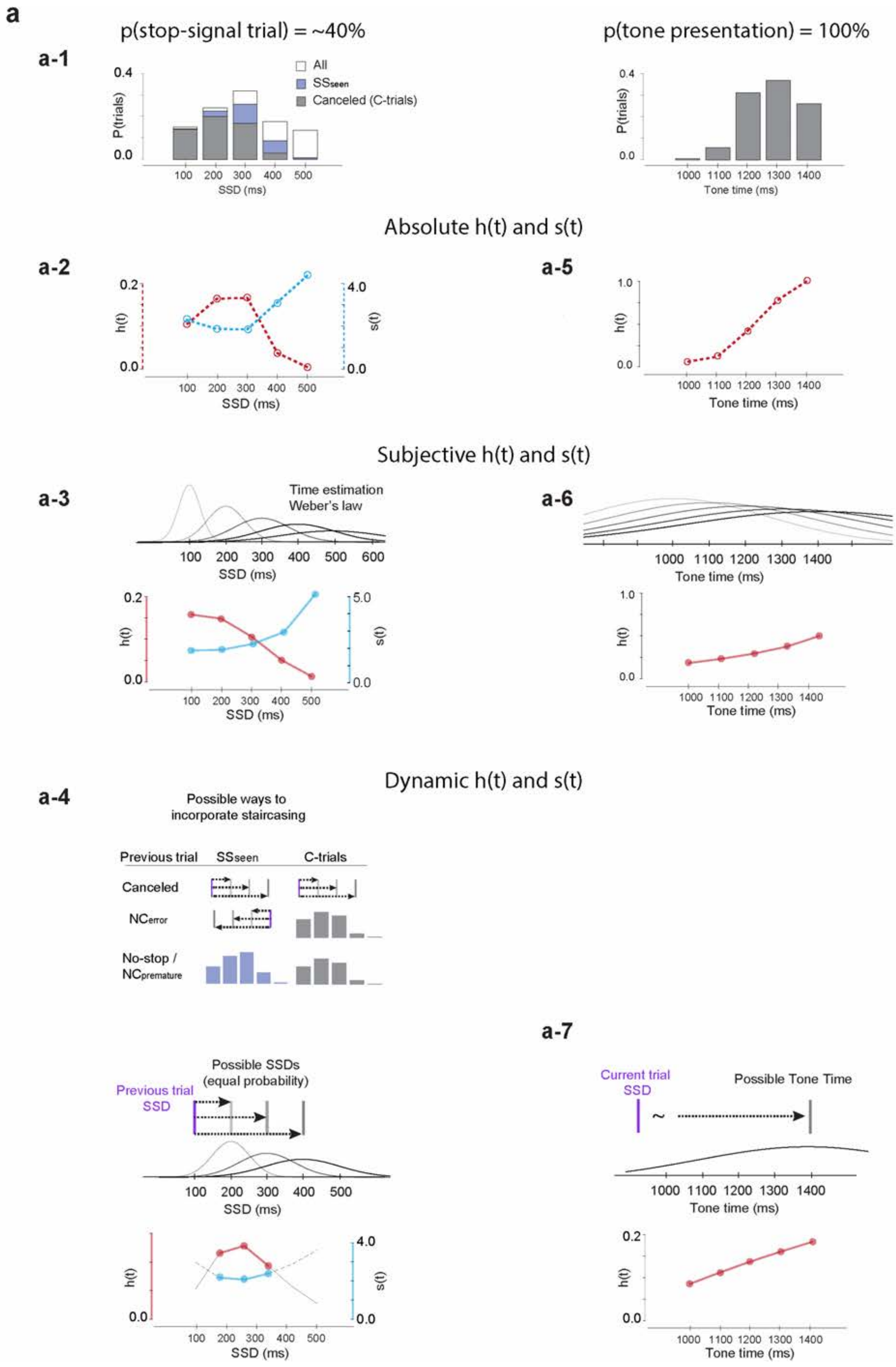

b Comparison between Conflict model and other models tested for post-SSRT facilitation, N2, and P3

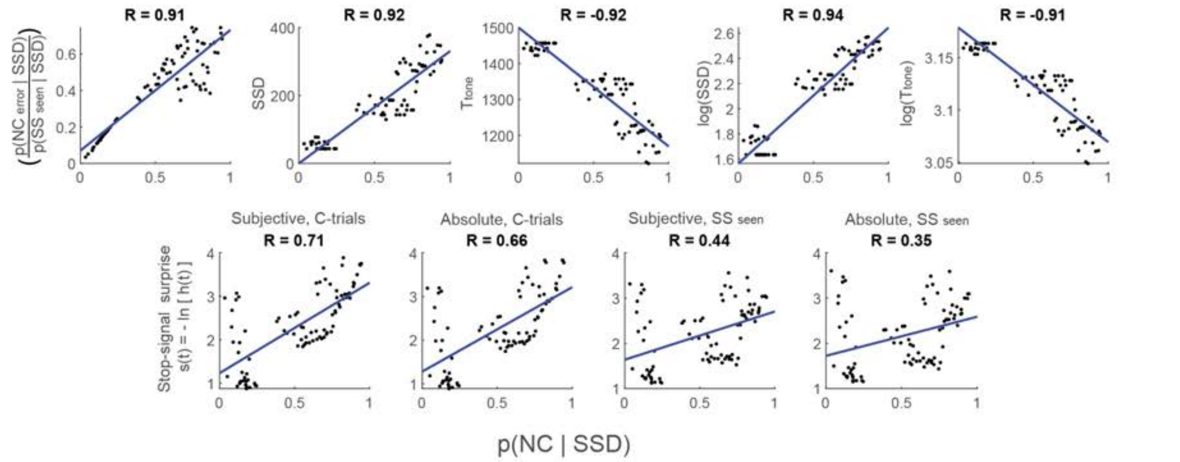

Comparison between S(t) Subjective, SSseen model and other models tested for post-SSRT facilitation, N2, and P3

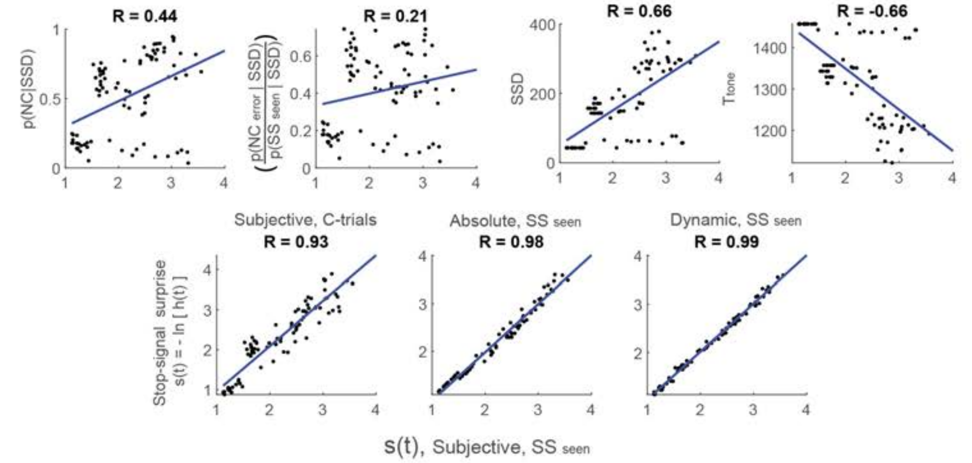

Comparison between log(SSD) model and other models tested for pre-SSRT activity

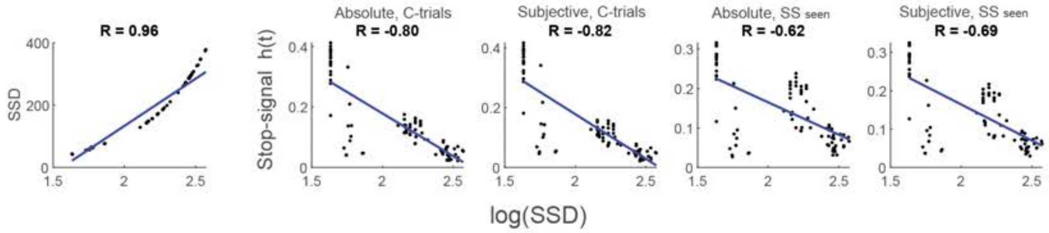

Comparison between log(T_{tone}) model and other models tested for pre-Tone activity

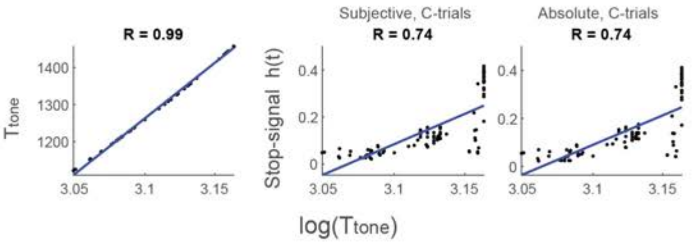

a, Patterns of neural spiking were analyzed in relation to multiple hazard rate (expectancy), and surprise measures. Stop-signals were scheduled to be presented at a range of times (**a-1,** left, white bars). At most times the stop-signal was seen (SS_{seen}, blue bars), which included canceled trials (gray bars) and explicit error noncanceled trials with RT > SSD (NC_{error}). By design, on canceled trials, the interval between presentation of the visual target and of the feedback tone was fixed at 1,500 ms. Thus, the interval from stop-signal to success tone (T_{tone}) was just T_{tone} = 1500 – SSD (**a-1,** right). Monkeys can learn temporal regularities to form moment-by-moment expectations for task events. Neural responses can encode this expectation prospectively, before the event, or surprise about violation of these expectations, retrospectively, after the event. Expectation was operationalized by hazard rate. Surprise was calculated as the Shannon Information, derived from the hazard rate. Guided by previous research [9-13], three kinds of hazard rate were calculated based on different assumptions: (i) the absolute hazard rate, (ii) subjective hazard rate, and (iii) dynamic hazard rate.

Absolute hazard rate was derived from the distribution of time (t) values as h(t) = f(t) / [1-F(t)], where f(t) is the density distribution and F(t) is the cumulative distribution. Monkeys can gain knowledge only from SSD that are experienced, so we used only trials in which stop-signal was seen before the response (SS_{seen} trials). We also considered the possibility that the knowledge from task structure is gained in a trial-specific manner, only restricted to canceled trials (C-trials). Now, because stop-signal presentation was probabilistic with ~40% likelihood, the hazard function was calculated with the conditional probability of stop-signal appearance for each session. This conditional probability accounted for the fact that on a given trial, because of the uncertainty associated with the current trial being a stop-signal trial, if time passes and no stop-signal is observed, the belief about the current trial being a stop-signal trial drops, reducing the expectation for stop-signal appearance. Consequently, conditional hazard rate does not approach 1.0 and is not monotonic. Because our approach relied on mean responses across many trials having different patterns of preceding trial history within each SSD bin, we assumed variations in the inferred state only change due to passage of time within a trial. A conditional probability was not necessary for the hazard rate of T_{tone} because the feedback tone occurred with 100% certainty. Representative plots of the absolute hazard rates of SSD and T_{tone} are shown in a-2 and a-5 (red) and the associated surprise for SSD is shown in a-2 (blue). The quantities shown here are based on C-trials, corresponding to $h(t)_{\text{Absolute, C-trials}}$ and $s(t)_{\text{Absolute, C-trials}}$. We did not analyze the period after the tone, therefore surprise for tone was not considered.

Subjective hazard rate was derived from time (t) values that incorporated the imprecision in time perception. The estimation of elapsed time has increasing uncertainty over longer intervals [14]. To account for this imprecision, f(t) was convolved with a Gaussian kernel with standard distribution proportional to the elapsed time:

$$g(t) = \frac{1}{\emptyset t\sqrt{2\pi}} \int_{-\infty}^{\infty} f(t) e^{-(\frac{\text{T-t}}{\sqrt{2}\emptyset t})^2} dx$$

The coefficient of variation, ø, was set to 0.26 based on previous research [11, 14] (**a-3, a-6,** top). Representative plots of the subjective hazard rates of SSD and T_{tone} are shown in **a-3** and **a-6** (red) with associated surprise for SSD in **a-3** (blue). The quantities shown here are based on C-trials, thus corresponding to $h(t)_{\text{Subjective, C-trials}}$ and $s(t)_{\text{Subjective, C-trials}}$.

Dynamic hazard rate was derived from time values incorporating both imprecision in time estimation and the restricted range of possible SSD or T_{tone} values based on knowledge of regularities in the preceding intervals experienced [12]. This was important because to ensure that monkeys failed to cancel on ~50% of all stop-signal trials, SSD was adapted dynamically in a staircase algorithm. After noncanceled trials, SSD was decreased. After canceled trials, SSD was increased. To discourage monkeys from exploiting the stair-casing algorithm, SSD was adjusted in steps of 1, 2, or 3 intervals randomly selected with uniform 1/3 probability. Nevertheless, we explored the possibility that monkeys can incorporate this knowledge to predict the timing of the upcoming SSD. A prediction of SSD would influence estimation of T_{tone} because T_{tone} = 1500 – SSD. The left panel of **a-4** portrays how the preceding trial restricted possible SSD values in the current trial. Separate calculations were done with C-trials and SS_{seen} trials. For $h(t)_{\text{Dynamic, C-trials}}$ stair-casing knowledge was only incorporated following canceled trials. For $h(t)_{\text{Dynamic, SSseen}}$ stair-casing knowledge was incorporated for both canceled trials and NC_{error} trials. For all other trials, absolute f(t) was used. Representative plots of the subjective hazard rates of SSD and T_{tone} are shown in **a-4** and **a-7** (red, $h(t)_{\text{Dynamic, C-trials}}$) with associated surprise for SSD (blue, $s(t)_{\text{Dynamic, C-trials}}$). The gray traces in lower panel of a-4 corresponds to the calculated h(t) and s(t), and the colored portion of the trace corresponds to possible values at the SSDs restricted by the staircasing algorithm.

b, Some behavioral and task measures were correlated, but random and systematic variations within and across sessions enabled differentiation. The relationship of neural activity before SSRT and before the tone to each of the behavioral and task measures was compared In a set of mixed-effects models using Bayesian Information Criteria (BIC) [15]. Top two rows of panels plot numerical relationships between indicated measures (y-axis) and p(NC | SSD). Four of the six surprise models are shown. Third and fourth rows plot relationships between indicated measures and surprise $s(t)_{\text{subjective, SS seen}}$. Fifth row plots relationships of SSD and four of six stop-signal hazard rate measures to log(SSD). The bottom row plots the relationships of T_{tone} and two of three hazard rate measures to log(T_{tone}).

Supplementary Figure 4 | Conflict Neurons.

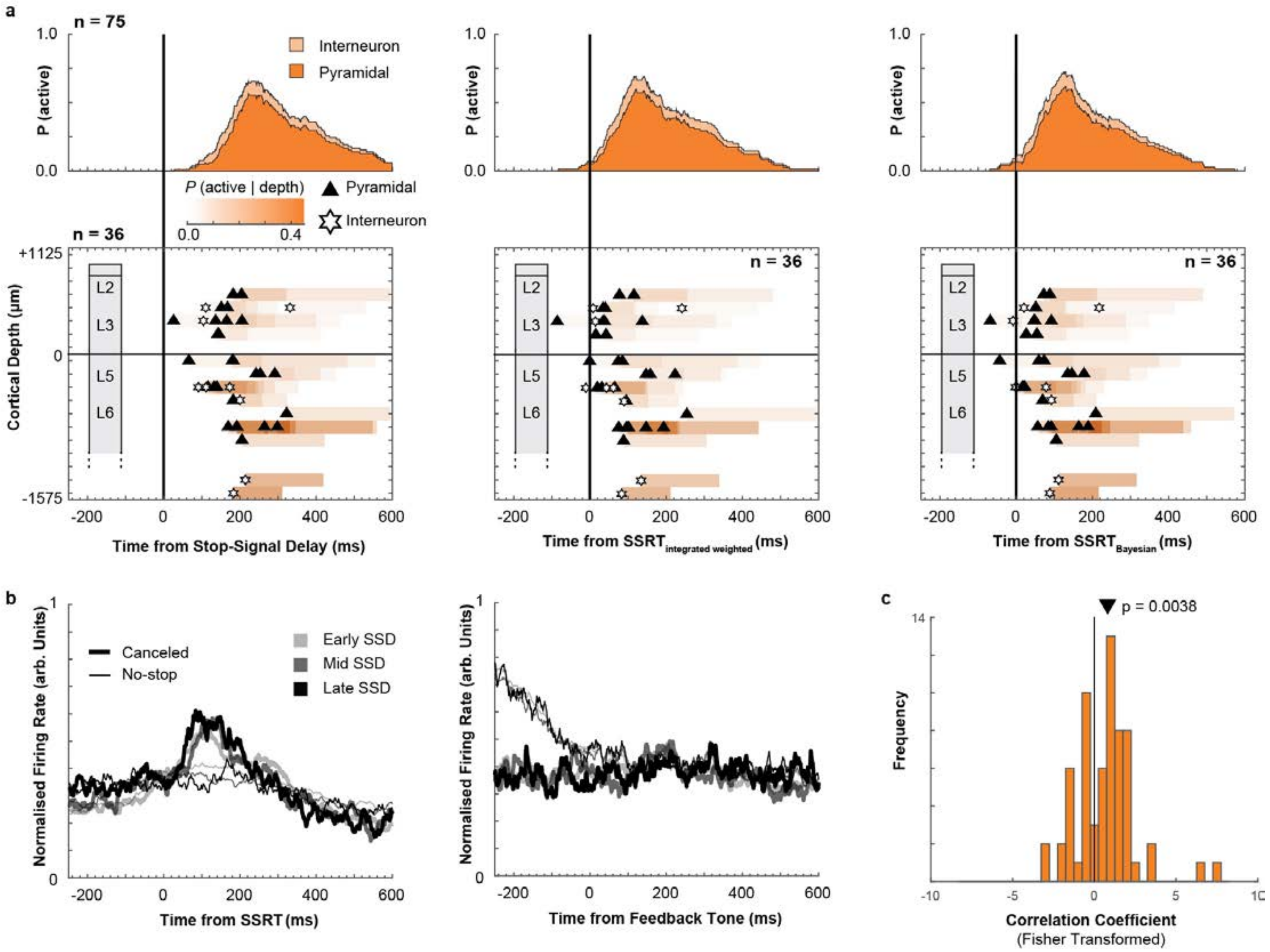

a, Recruitment and time-depth plots aligned on presentation of the stop-signal (left), SSRT calculated by the method of integration (middle, same as **Fig 3**), and SSRT calculated by the Bayesian Estimation of Ex-Gaussian Stop-Signal reaction time (BEESTS) (right). The patterns supporting the conclusions do not vary with the method of alignment. This neural signal nearly exclusively follows SSRT.

b, Normalized population spike-density functions aligned on SSRT (left) and feedback time (right) for canceled (thick) and latency-matched no-stop-signal trials (thin) subsampled from trials with low (lightest), middle (intermediate), and high (darkest) p(NC | SSD). The scaling of the modulation with SSD and p(NC | SSD) is evident across the sample. Note that the activity of neurons aligned on tone, on no-stop-signal trials during the pre-tone interval can be larger than that on canceled trials because of post-saccadic activity bleeding into this time period.

c, Distribution of correlation coefficients of post-SSRT modulation as a function of p(NC | SSD) across neurons. Correlation coefficients were transformed into a normal distribution using Fisher-transformation. The distribution was shifted significantly positively (Two-tailed Wilcoxon test, p = 0.004). The black triangle indicates the mean of the distribution. This plotting format clarifies the correspondence between the modulation patterns of SEF and of brainstem dopamine neurons of monkeys performing saccade countermanding tasks [5].

Supplementary Figure 5 | Event-Timing Neurons.

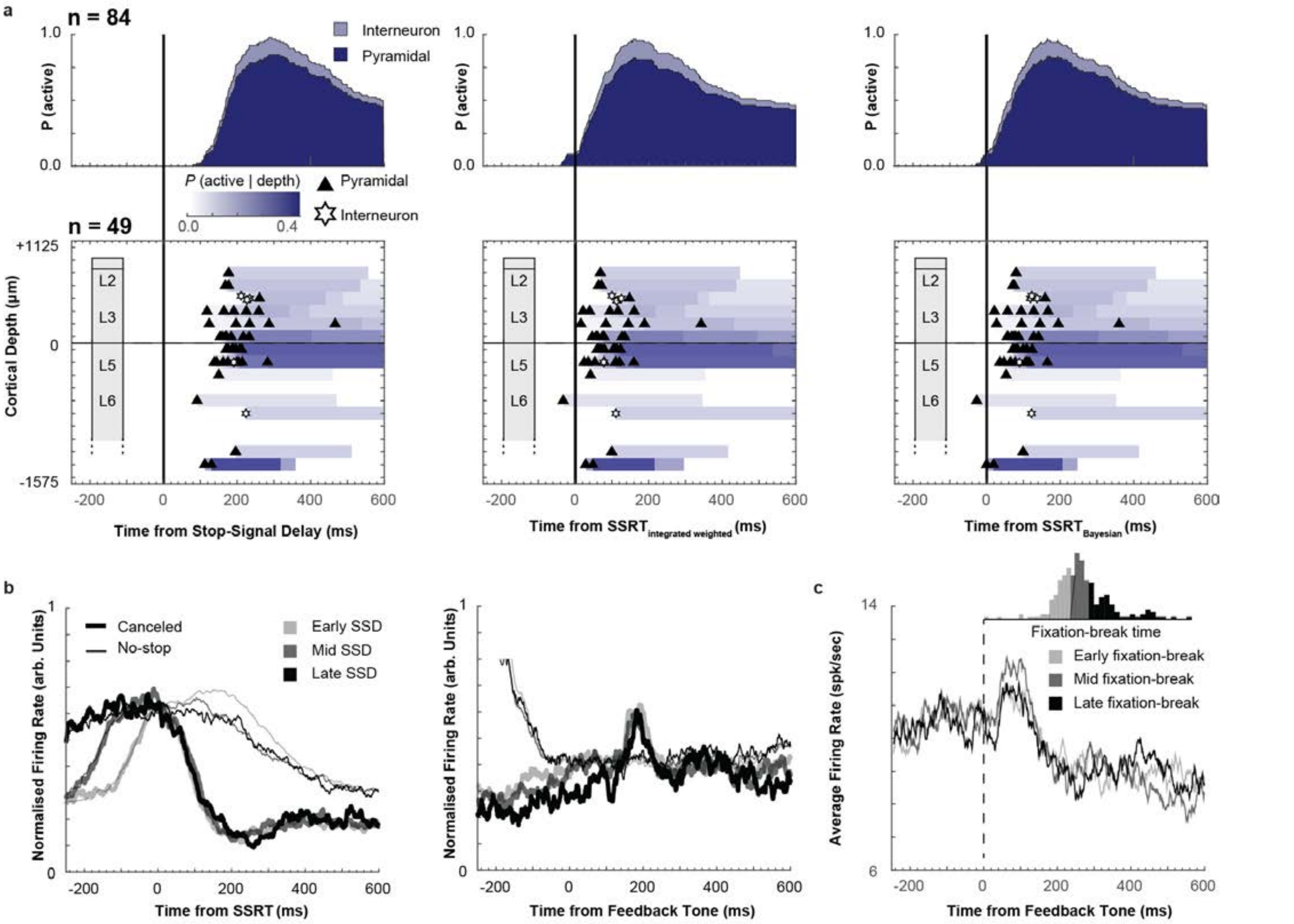

a, Recruitment and time-depth plot aligned on stop-signal (left), SSRT calculated based on the method of integration (middle, same as **Fig 4**), and SSRT by BEESTS (right). The patterns supporting the conclusions reported in the main text do not vary with the method of alignment. This neural signal nearly exclusively follows SSRT and persists until the tone.

b, Normalized population spike-density functions aligned on SSRT (left) and feedback time (right) for canceled (thick) and latency-matched no-stop-signal trials (thin) subsampled from trials with low (lightest), middle (intermediate), and high (darkest) SSD. The timing of the modulation across SSD is evident across the sample. Note that the activity of neurons aligned on tone, on no-stop-signal trials during the pre-tone interval can be larger than that on canceled trials because of post-saccadic activity bleeding into this time period.

c, Average spike-density function for subset of neurons (n = 84) aligned on feedback tone for short (light gray), medium (dark gray), and long (black) periods until monkeys shifted gaze from the central point or blinked (inset plots distribution of the time of the first saccade or blink following the feedback tone in one session). The temporal dynamics of the tone-aligned activity did not depend on the time at which fixation was broken following the feedback tone. This pattern was observed on almost all individual neurons (not shown).

Supplementary Figure 6 | Goal Maintenance Neurons

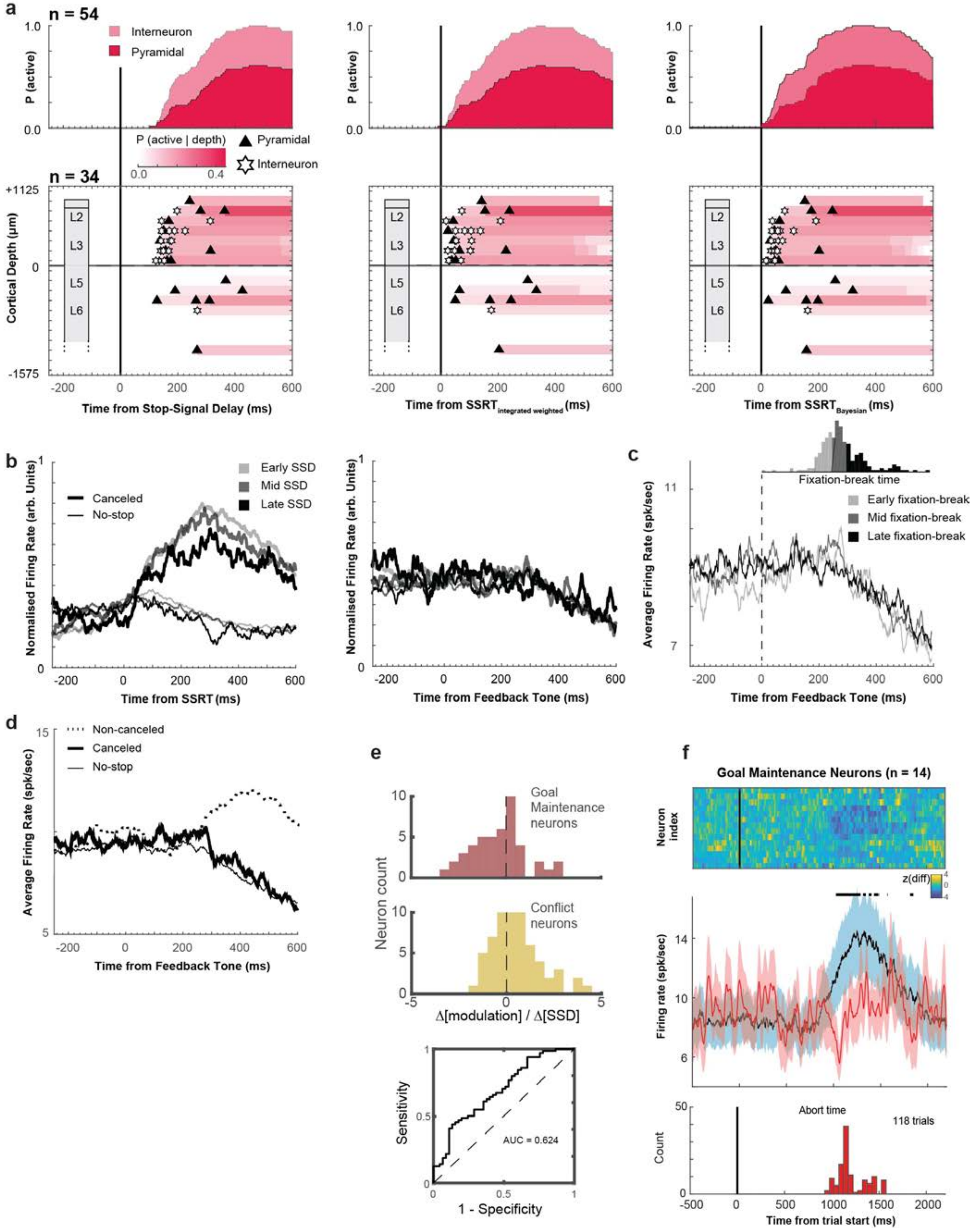

a, Recruitment and time-depth plots aligned on stop-signal (left), SSRT calculated based on the method of integration (middle, same as **Fig 5**), and SSRT by BEESTS (right). The patterns supporting the conclusions reported in the main text do not vary with the method of alignment. This neural signal grows after SSRT and persists until after the tone.

b, Normalized population spike-density functions aligned on SSRT (left) and feedback time (right) for canceled (thick) and latency-matched no-stop-signal trials (thin) subsampled from trials with low (lightest), middle (intermediate), and high (darkest) SSD. The scaling of the modulation across SSD is evident across the sample.

c, Average spike-density function for subset of neurons (n = 54) aligned on feedback tone for short (light gray), medium (dark gray), and long (black) periods until monkeys shifted gaze from the central point or blinked (inset plots distribution of the time of the first saccade or blink following the feedback tone in one session). The temporal dynamics of the tone-aligned activity did not depend on the time at which fixation was interrupted following the feedback tone. This pattern was observed on almost all individual neurons (not shown).

d, Population spiked-density function on canceled (thick solid), no-stop-signal (thin), and noncanceled (thick dashed) trials. A large proportion of Goal Maintenance neurons were also classified as Loss neurons, with higher activity on unrewarded compared to rewarded trials.

e, Rate of change of spiking as a function of SSD for Goal Maintenance (red) and Conflict (yellow) neurons. With longer SSD, Goal Maintenance neurons tended to be less active, but Conflict neurons were more active. The distinction between Goal Maintenance and Conflict neurons on this measure is confirmed in the ROC plot.

f, Goal Maintenance neurons with reduced activity when fixation was interrupted. The contrast of neural spiking between trials in which reward was delivered after saccades were canceled and trials when fixation was interrupted by a saccade or a blink before the feedback tone. Matching trials for SSD, 14/54 Goal Maintenance neurons had at least 5 aborted trials. The difference across conditions is plotted as a heat-map (top) with one neuron per row and average ± SEM spike density (middle) for successful canceled trials (black) and aborted trials (red), smoothed for visualization purposes, with periods of significant difference (two-tailed Wilcoxon test, $p < 0.05$, no multiple comparison correction) indicated by black horizontal lines above the SDF plots. The histogram plots the abort times.

Supplementary Figure 7 | N2/P3 Characteristics.

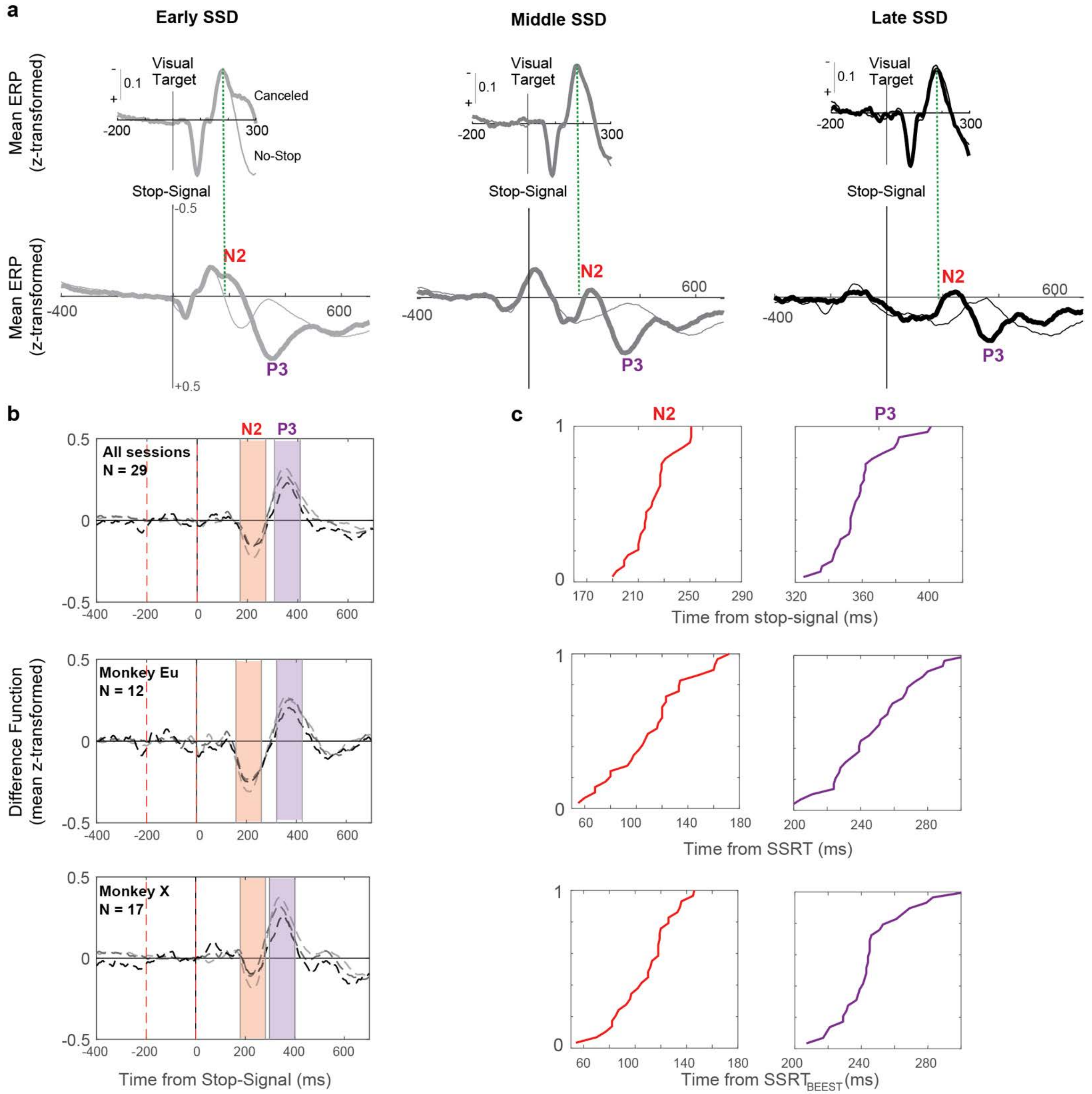

a, ERP aligned on target presentation (top), and stop-signal (bottom) for early (left), intermediate (middle), and late (right) SSD. At all SSD, the visually-evoked negativity (green dashed line) occurs earlier than the peak of N2 by at least 40 ms. Thus, the N2 is not just a visual response.

b, N2 and P3 difference plots for all sessions (top) and sessions from monkey Eu (middle) and X (bottom) with ±50 ms period around the peak shaded. The N2 and P3 exhibited similar features across monkeys. **c,** Cumulative distributions of N2 (left) and P3 (right) peak times aligned on stop-signal (top), SSRT by method of integration (middle) and SSRT by BEESTS (bottom). The distributions are narrower when aligned on stop-signal.

Supplementary Figure 8 | N2/P3 relationship with spiking.

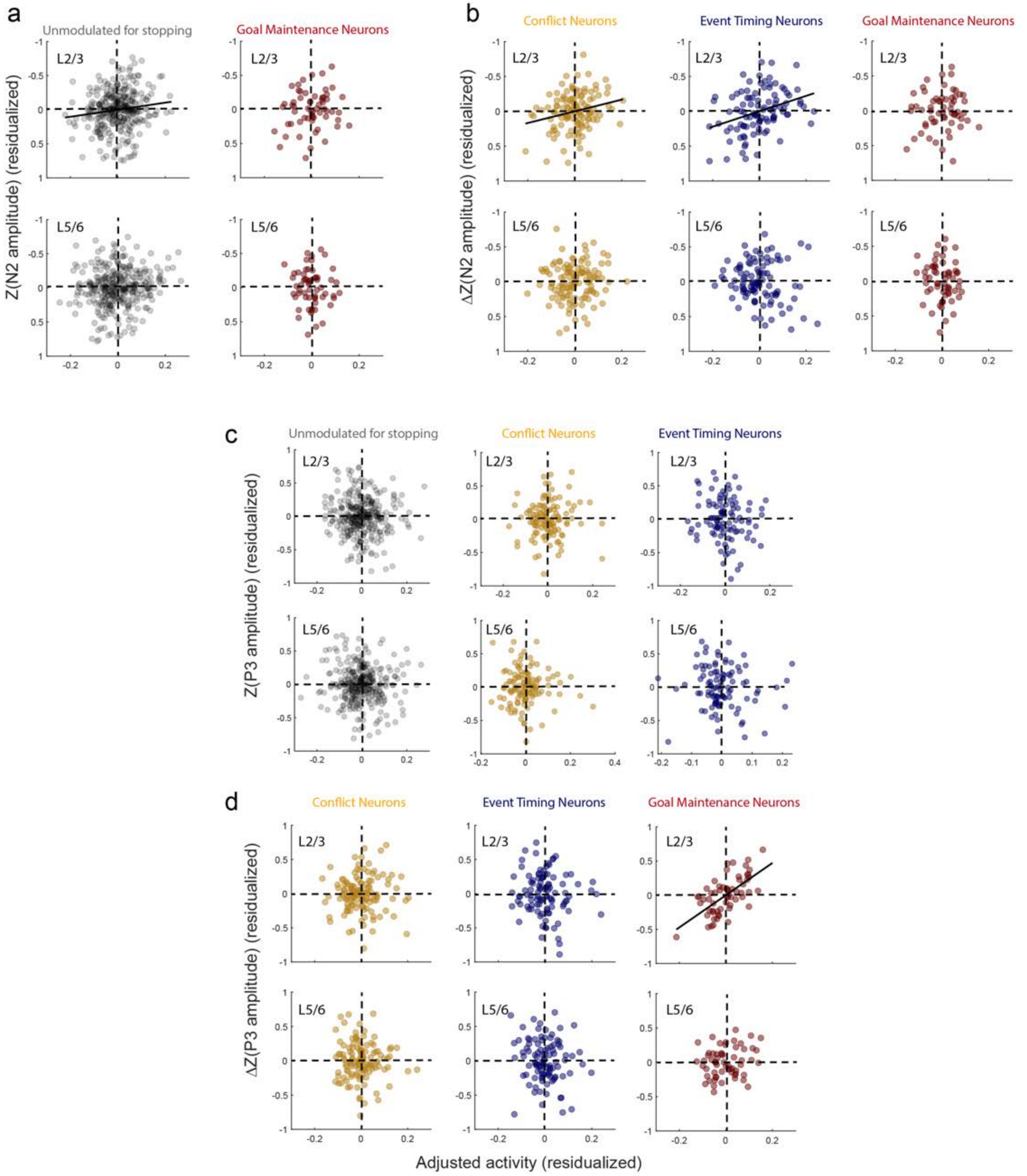

a, N2 polarization was predicted by the spiking of SEF neurons that were not modulated around the time of SSRT in L2/3 (Multiple Linear regression (two-tailed) with L2/3 and L5/6 activity as predictors, $t(317) = -2.51$, $p = 0.012$) but not L5/6 (left) but was unrelated to the spiking of Goal Maintenance neurons in L2/3 or L5/6 (right).

b, Difference of N2 polarization between canceled and latency-matched no-stop-signal trials was predicted by spiking of Conflict ($t(117) = -2.63$, $p = 0.010$) and Event timing neurons in L2/3 ($t(97) = -3.57$, $p < 0.001$) but not L5/6 and not by spiking of Goal maintenance neurons.

c, P3 polarization was unrelated to the spiking of SEF unmodulated neurons, Conflict neurons, or Event timing neurons in L2/3 or L5/6.

d, Difference of P3 polarization between canceled and latency-matched no-stop-signal trials was not predicted by spiking of Conflict or Event timing neurons but was predicted by the spiking of Goal maintenance neurons in L2/3 ($t(57) = 6.26$, $p < 0.001$) but not in L5/6. The statistics for the Linear regression models for Conflict, Goal Maintenance, and Event Timing neurons are presented in **Supplementary Tables 3, 4, and 5**.

Supplementary Figure 9 | Relationship of SEF modulation to substantia nigra pars compacta and caudate nucleus.

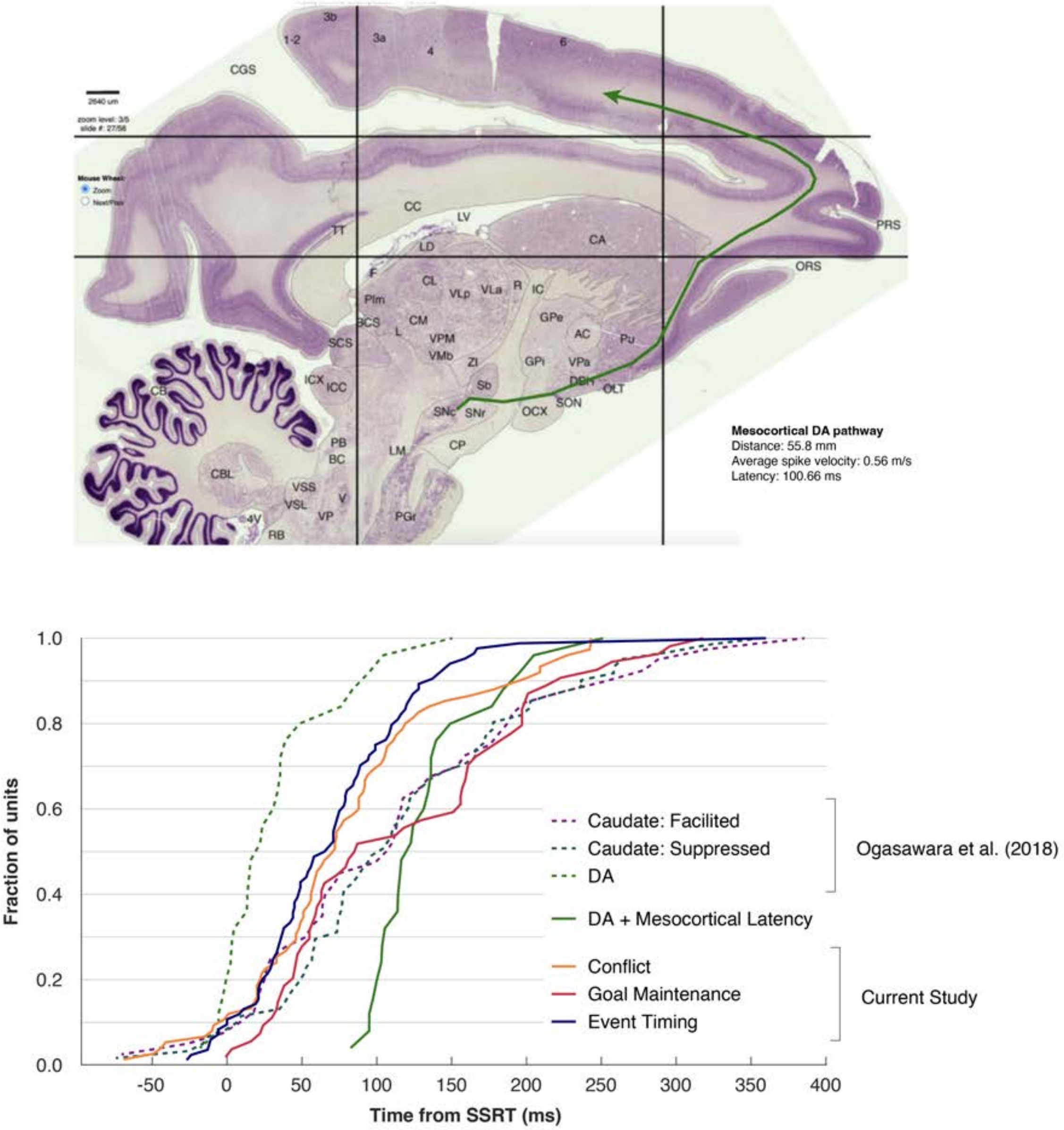

a, The mesocortical pathway of macaque monkeys. To infer the temporal relationship between VTA/SNpc and SEF signals, the mesocortical pathway was traced on a sagittal slice from the RH12 dataset (Dataset 6, RH12, Slide 27/56, Sagittal, Nissl; brainmaps.org). The estimated was 55.8 mm. Based on measurements of the conduction velocity of DA axons in rodents and assuming similar values for primates (0.54 m/s [16]; 0.55 m/s [17, 18]; 0.58 m/s [19]), we estimate that the conduction time of a spike from SNpc to SEF is 100.7 ms (average of 103.4 ms, 101.5 ms, 101.5 ms, and 96.3 ms, respectively).

b, Modulation times of the SEF neurons and of dopaminergic and striatal neurons. Cumulative distributions for Conflict (gold), Event Timing (blue), and Goal Maintenance (red) neurons in SEF are plotted with the latencies of dorsolateral SNpc neurons (dashed green) and of facilitated (dashed purple) and suppressed (dashed blue) neurons in the head of the caudate nucleus obtained from [5]. Also plotted is the estimated distribution of DA spike arrival times in SEF based on the conduction time from SNpc to SEF (green).

Supplementary Table 1 | Sampling distributions across recording sites and cortical depth.

Site	Number penetrations	All neurons	Conflict	Event Timing	Goal Maintenance
np1	6	140	10	29	12
np2	7	142	29	6	8
P1	6	104	12	41	1
P2	6	133	18	6	30
P3	4	56	6	2	3
Total count		575	75	84	54
$X^2(4, 575)$	test statistics	-	11.62	84.13	39.3
	p-value	-	0.020	< 0.001	< 0.001

Layer		All neurons	Conflict	Event Timing	Goal Maintenance
L2		34	2	3	7
L3		114	11	22	19
L5		88	14	19	6
L6		39	7	2	1
L6+		18	2	3	1
Total count		293	36	49	34
$X^2(4, 293)$	test statistics	-	4.28	7.33	11.24
	p-value	-	0.369	0.120	0.024

Layer	All neurons	Event Timing with preTone ramping
L2/upperL3	90	2
Lower L3/ L5	146	20
L6/L6+	57	3
Total count	293	
$X^2(2, 293)$ test statistics	-	10.37
p-value		0.006

Chi-square test for homogeneity was computed for each signal in a 5 (sites or layers) by 2 (with or without the signal) contingency matrix based on the counts of units.

Top, Count of Conflict, Event Timing, and Goal Maintenance signals across the 575 neurons sampled from five sites in monkey Eu and monkey X. Three of the five sites were sampled with perpendicular penetrations (Eu: P1, X: P2 and P3) and two were not (Eu: np1, X: np2). The probability of sampling each kind of signal varied significantly across sites.

Middle, Count of the neural signals from sites P1, P2 and P3 across cortical depth. Contacts ranged from depth 1 (shallowest) to depth 19 (deepest) [1, 2]. Neurons were assigned to the four cellular cortical layers plus contacts located beyond the L6 boundary. Conflict and Event timing neurons were distributed homogeneously across depth. Goal Maintenance neurons were significantly more concentrated in L2/3 relative to L5/6.

Bottom, Chi-square test of laminar homogeneity for Event Timing neurons with pre-tone ramping showing that these neurons were significantly more clustered in lower L3 and L5 (Fig 3b). The boundary between upper and lower L3 and between L5 and L6 are shown in Fig 3b (horizontal dashed lines).

Supplementary Table 2 | List of considered models.

Model name	Quantity	Mathematical expression
Conflict	Probability of not canceling	p(NC | SSD)
Error-Likelihood	Probability of not canceling despite seeing the stop-signal	$p(NC_{error} \mid SSD) / p(SS_{seen} \mid SSD)$
Time-based	Stop-signal delay	SSD<br>log(SSD)
	Feedback tone time	T_{tone} = 1500 - SSD<br>$\log(T_{tone})$

			Model variants			
			Trials used for learning time distribution	Additional task knowledge	Time perception inaccuracy	Descriptor
Hazard Rate	Stop-signal hazard rate	h(t) = f(t) / [1 - F(t)]	canceled trials	--	--	$h(t)_{\text{Absolute, C-trials}}$
			canceled trials	--	Weber's law	$h(t)_{\text{Subjective, C-trials}}$
			canceled trials	SSD staircasing	Weber's law	$h(t)_{\text{Dynamic, C-trials}}$
			SS seen trials	--	--	$h(t)_{\text{Absolute, SS seen}}$
			SS seen trials	--	Weber's law	$h(t)_{\text{Subjective, SS seen}}$
			SS seen trials	SSD staircasing	Weber's law	$h(t)_{\text{Dynamic, SS seen}}$
	Feedback Tone hazard rate		canceled trials	--	--	$h(t)_{\text{Absolute, C-trials}}$
			canceled trials	--	Weber's law	$h(t)_{\text{Subjective, C-trials}}$
			canceled trials	$T_{tone} = 1500 - SSD_{\text{current trial}}$	Weber's law	$h(t)_{\text{Dynamic, C-trials}}$
Surprise	Stop-signal surprise	$s(t) = -\log_2[h(t)]$	canceled trials	--	--	$s(t)_{\text{Absolute, C-trials}}$
			canceled trials	--	Weber's law	$s(t)_{\text{Subjective, C-trials}}$
			canceled trials	SSD staircasing	Weber's law	$s(t)_{\text{Dynamic, C-trials}}$
			SS seen trials	--	--	$s(t)_{\text{Absolute, SS seen}}$
			SS seen trials	--	Weber's law	$s(t)_{\text{Subjective, SS seen}}$
			SS seen trials	SSD staircasing	Weber's law	$s(t)_{\text{Dynamic, SS seen}}$

The nomenclature, mathematical expression, and descriptor for different models and their variants are shown. Hazard rate and surprise models had different variants based on different assumptions indicated in the table (see **Supplementary Figure 3a**).

Supplementary Table 3 | Comparison of model fits for Conflict neurons exhibiting post-SSRT facilitation

Signal	Response Period	BIC	Δ BIC	Predictor (Z-scaled)	df	β	SE	t	p
**Conflict**	Transient post-SSRT	**980.10**	**0**	**intercept**	**212**	**2.61**	**0.20**	**13.20**	**< 0.001**
				**p(NC | SSD)**		**0.61**	**0.14**	**4.24**	**< 0.001**
		983.59	3.52	intercept	212	2.59	0.20	13.30	< 0.001
				$p(NC_{error} \| SSD) / p(SS_{seen} \| SSD)$		0.54	0.15	3.72	< 0.001
		981.40	1.32	intercept	212	2.61	0.20	13.22	< 0.001
				SSD		0.59	0.14	4.06	< 0.001
		982.23	2.15	intercept	212	2.60	0.20	13.27	< 0.001
				log(SSD)		0.56	0.14	3.93	< 0.001
		981.40	1.32	intercept	212	2.61	0.20	13.22	< 0.001
				T_{tone}		-0.59	0.14	-4.06	< 0.001
		981.68	1.61	intercept	212	2.61	0.20	13.20	< 0.001
				$log(T_{tone})$		-0.59	0.15	-4.03	< 0.001
		980.86	0.78	intercept	212	2.63	0.20	13.45	< 0.001
				$s(t)_{absolute,\ C\text{-}trials}$		0.66	0.16	4.11	< 0.001
		981.56	1.49	intercept	212	2.63	0.20	13.41	< 0.001
				$s(t)_{subjective,\ C\text{-}trials}$		0.63	0.16	4.02	< 0.001
		982.12	2.04	intercept	212	2.62	0.20	13.38	< 0.001
				$s(t)_{dynamic,\ C\text{-}trials}$		0.63	0.16	3.94	< 0.001
		988.81	8.74	intercept	212	2.60	0.20	13.28	< 0.001
				$s(t)_{absolute,\ SS\ seen}$		0.48	0.17	2.88	0.0043
		986.85	6.77	intercept	212	2.60	0.20	13.29	< 0.001
				$s(t)_{subjective,\ SS\ seen}$		0.52	0.16	3.23	0.0014
		985.76	5.69	intercept	212	2.61	0.20	13.32	< 0.001
				$s(t)_{dynamic,\ SS\ seen}$		0.56	0.16	3.40	< 0.001

Statistical outcomes are given for mixed-effects modelling identifying which model best fits the neural modulation of Conflict neurons and behavioral/task parameters. The statistics for the best-fit model (top row, bold text) and competing models (lower rows) are shown. The mixed-effects model allowed for modeling random intercepts grouped by neuron for spiking measures. No consideration for multiple comparison is included in these statistics. This data contributes to the statistics table in Fig. 3d.

Supplementary Table 4 | Comparison of model fits for Event Timing neurons

Signal	Response Period	BIC	Δ BIC	Predictor (Z-scaled)	df	β	SE	t	p
Event Timing	pre-SSRT activity	**1682.60**	**0**	**intercept**	**250**	**16.19**	**1.28**	**12.62**	**< 0.001**
				**log(SSD)**		**0.76**	**0.24**	**3.24**	**0.001**
		1685.35	2.71	intercept	250	16.19	1.28	12.61	< 0.001
				SSD		0.66	0.24	2.77	0.006
		1690.86	8.21	intercept	250	16.19	1.28	12.67	< 0.001
				$h(t)_{\text{absolute, C-trials}}$		-0.41	0.29	-1.41	0.158
		1689.73	7.08	intercept	250	16.19	1.28	12.68	< 0.001
				$h(t)_{\text{subjective, C-trials}}$		-0.51	0.29	-1.77	0.078
		1690.69	8.04	intercept	250	16.19	1.28	12.68	< 0.001
				$h(t)_{\text{dynamic, C-trials}}$		-0.45	0.30	-1.47	0.142
		1692.80	10.15	intercept	250	16.19	1.28	12.63	< 0.001
				$h(t)_{\text{absolute, SS seen}}$		-0.08	0.35	-0.22	0.823
		1691.79	9.14	intercept	250	16.19	1.28	12.68	< 0.001
				$h(t)_{\text{subjective, SS seen}}$		-0.37	0.35	-1.03	0.303
		1691.60	8.95	intercept	250	16.19	1.28	12.68	< 0.001
				$h(t)_{\text{dynamic, SS seen}}$		-0.38	0.34	-1.12	0.264
Event Timing	pre-Tone activity	**678.20**	**0**	**intercept**	**112**	**12.77**	**1.79**	**7.15**	**< 0.001**
				**$\log(T_{tone})$**		**0.67**	**0.20**	**3.41**	**< 0.001**
		678.29	0.01	intercept	112	12.77	1.79	7.15	< 0.001
				T_{tone}		0.67	0.20	3.41	0.001
		681.28	3.00	intercept	112	12.77	1.78	7.16	< 0.001
				$h(t)_{\text{absolute, C-trials}}$		0.58	0.20	2.87	0.005
		682.34	4.06	intercept	112	12.77	1.81	7.05	< 0.001
				$h(t)_{\text{subjective, C-trials}}$		1.27	0.47	2.68	0.008
		682.69	4.41	intercept	112	12.77	1.81	7.04	< 0.001
				$h(t)_{\text{dynamic, C-trials}}$		1.24	0.47	2.61	0.010

Statistical outcomes are given for mixed-effects modelling identifying which model best fits the ramping neural modulation of Event-Timing neurons and behavioral/task parameters. The statistics for the best-fit model for pre-SSRT and pre-Tone activity (upper row, bold text) and respective competing models (lower rows) are shown. The mixed-effects model allowed for modeling random intercepts grouped by neuron for spiking measures. No consideration for multiple comparison is included in these statistics. This data contributes to the statistics tables in Fig. 4d and 4g.

Supplementary Table 5 | Comparison of model fits for Goal Maintenance neurons exhibiting post-SSRT facilitation

Signal	Response Period	BIC	Δ BIC	Predictor (Z-scaled)	df	β	SE	t	p
**Goal Maintenance**	Sustained post-SSRT		**0**	**intercept**	**151**	**6.52**	**0.73**	**8.87**	**< 0.001**
				**$s(t)_{\text{subjective, SS seen}}$**		**-0.65**	**0.17**	**-3.91**	**< 0.001**
		801.67	4.10	intercept	151	6.54	0.74	8.89	< 0.001
				p(NC | SSD)		-0.48	0.15	-3.26	0.0013
		805.73	8.16	intercept	151	6.56	0.74	8.90	< 0.001
				$p(NC_{error} \| SSD) / p(SS_{seen} \| SSD)$		-0.38	0.15	-2.50	0.013366
		799.45	1.88	intercept	151	6.54	0.73	8.91	< 0.001
				SSD		-0.53	0.15	-3.62	< 0.001
		800.55	2.98	intercept	151	6.55	0.73	8.91	< 0.001
				log(SSD)		-0.50	0.14	-3.45	< 0.001
		799.45	1.88	intercept	151	6.54	0.73	8.91	< 0.001
				T_{tone}		0.53	0.15	3.62	< 0.001
		799.37	1.80	intercept	151	6.54	0.73	8.91	< 0.001
				$\log(T_{tone})$		0.53	0.15	3.63	< 0.001
		798.52	0.95	intercept	151	6.52	0.73	8.87	< 0.001
				$s(t)_{\text{absolute, C-trials}}$		-0.59	0.16	-3.71	< 0.001
		799.02	1.45	intercept	151	6.52	0.73	8.88	< 0.001
				$s(t)_{\text{subjective, C-trials}}$		-0.58	0.16	-3.77	< 0.001
		798.89	1.32	intercept	151	6.52	0.73	8.88	< 0.001
				$s(t)_{\text{dynamic, C-trials}}$		-0.61	0.16	-3.84	< 0.001
		798.47	0.90	intercept	151	6.51	0.74	8.86	< 0.001
				$s(t)_{\text{absolute, SS seen}}$		-0.65	0.17	-3.76	< 0.001
		798.00	0.43	intercept	151	6.52	0.74	8.86	< 0.001
				$s(t)_{\text{dynamic, SS seen}}$		-0.62	0.17	-3.69	< 0.001

Statistical outcomes are given for mixed-effects modelling identifying which model best fits the neural modulation of Goal Maintenance neurons and behavioral/task parameters. The statistics for the best-fit model (top row, bold text) and competing models (lower rows) are shown. The mixed-effects model allowed for modeling random intercepts grouped by neuron for spiking measures. No consideration for multiple comparison is included in these statistics. This data contributes to the statistics table in Fig. 5d.

Supplementary Table 6 | Comparison of model fits for ERP N2 and P3 components.

Signal	Response Period	BIC		Predictor (Z-scaled)	df	β	SE	t	p
N2	post-SSRT	**-52.10**	**0**	**intercept**	**85**	**-0.120**	**0.023**	**-5.470**	**< 0.001**
				**$p(NC_{error}\|SSD) / p(SS_{seen} \| SSD)$**		**0.040**	**0.016**	**2.420**	**0.013**
		-49.75	2.88	intercept	85	-0.124	0.023	-5.397	< 0.001
				p(NC | SSD)		0.029	0.016	1.844	0.069
		-47.26	5.38	intercept	85	-0.124	0.024	-5.243	< 0.001
				SSD		0.014	0.016	0.912	0.364
		-47.56	5.07	intercept	85	-0.124	0.024	-5.215	< 0.001
				log(SSD)		0.017	0.016	1.072	0.28697
		-47.26	5.38	intercept	85	-0.124	0.024	-5.243	< 0.001
				T_{tone}		-0.014	0.016	-0.912	0.364
		-47.22	5.41	intercept	85	-0.124	0.024	-5.247	< 0.001
				$\log(T_{tone})$		-0.014	0.016	-0.894	0.374
		-49.57	3.06	intercept	85	-0.124	0.023	-5.286	< 0.001
				$s(t)_{absolute, C\text{-}trials}$		-0.005	0.017	-0.294	0.770
		-48.89	3.74	intercept	85	-0.124	0.024	-5.255	< 0.001
				$s(t)_{subjective, C\text{-}trials}$		-0.002	0.017	-0.128	0.898
		-48.78	3.85	intercept	85	-0.124	0.023	-5.372	< 0.001
				$s(t)_{dynamic, C\text{-}trials}$		-0.011	0.017	-0.626	0.533
		-46.51	6.12	intercept	85	-0.124	0.021	-5.828	< 0.001
				$s(t)_{absolute, SS\ seen}$		-0.035	0.018	-1.987	0.050
		-46.44	6.19	intercept	85	-0.124	0.022	-5.763	< 0.001
				$s(t)_{subjective, SS\ seen}$		-0.031	0.018	-1.767	0.081
		-46.77	5.86	intercept	85	-0.124	0.022	-5.750	< 0.001
				$s(t)_{dynamic, SS\ seen}$		-0.031	0.018	-1.727	0.088
P3	post-SSRT	**-94.60**	**0**	**intercept**	**85**	**0.230**	**0.02**	**10.60**	**< 0.001**
				**$\log(T_{tone})$**		**0.041**	**0.01**	**3.72**	**< 0.001**
		-91.73	2.88	intercept	85	0.231	0.022	10.697	< 0.001
				p(NC | SSD)		-0.037	0.011	-3.210	0.002
		-85.21	9.40	intercept	85	0.231	0.022	10.693	< 0.001
				$p(NC_{error}\|SSD) / p(SS_{seen} \| SSD)$		-0.022	0.012	-1.746	0.084
		-94.56	0.05	intercept	85	0.231	0.022	10.621	< 0.001
				SSD		-0.041	0.011	-3.717	< 0.001
		-93.02	1.59	intercept	85	0.231	0.022	10.698	< 0.001
				log(SSD)		-0.038	0.011	-3.441	< 0.001
		-94.56	0.05	intercept	85	0.231	0.022	10.621	< 0.001
				T_{tone}		0.041	0.011	3.717	< 0.001
		-88.45	6.16	intercept	85	0.231	0.022	10.639	< 0.001
				$s(t)_{absolute, C\text{-}trials}$		-0.033	0.012	-2.650	0.010
		-90.11	4.49	intercept	85	0.231	0.022	10.628	< 0.001
				$s(t)_{subjective, C\text{-}trials}$		-0.035	0.012	-2.869	0.005
		-89.92	4.68	intercept	85	0.231	0.022	10.612	< 0.001
				$s(t)_{dynamic, C\text{-}trials}$		-0.035	0.012	-2.836	0.006
		-88.84	5.77	intercept	85	0.231	0.022	10.585	< 0.001
				$s(t)_{absolute, SS\ seen}$		-0.035	0.013	-2.574	0.012
		-89.89	4.72	intercept	85	0.231	0.022	10.565	< 0.001
				$s(t)_{subjective, SS\ seen}$		-0.039	0.013	-2.924	0.004
		-89.71	4.89	intercept	85	0.231	0.022	10.571	< 0.001
				$s(t)_{dynamic, SS\ seen}$		-0.038	0.013	-2.885	0.005

Statistical outcomes are given for mixed-effects modelling identifying which model best fits the variation in the N2 and P3 components and behavioral/task parameters. The statistics for the best-fit model (top row, bold text) and competing models (lower rows) are shown. The mixed-effects model allowed for modeling random intercepts grouped by session for ERP measures. No consideration for multiple comparison is included in these statistics. This data contributes to the statistics table in Fig. 6c

Supplementary Table 7 | Multiplexed signals between Error, Gain, and Loss with Conflict, Event Timing, and Goal Maintenance.

			Conflict		Event Timing		Goal Maintenance	
**Signal type**	**N**	**Percent (%)**	**N**	**Percent (%)**	**N**	**Percent (%)**	**N**	**Percent (%)**
Error signal	61	10.6	10 (5)	13.3	25 (12)	29.8	4 (1)	7.4
Gain signal	91	15.8	11 (1)	14.7	24 (9)	28.6	4 (0)	7.4
Loss signal	189	32.9	30 (4)	40.0	14 (3)	16.7	33 (1)	61.1
Other	234	40.7	32	42.7	33	39.3	15	27.8
Total Count	575		75		84		54	
$X^2(3, N = 575)$		test statistics	1.02		44.86		19.43	
		p-value	0.791		< 0.001		< 0.001	

Count of Conflict, Event Timing, and Goal Maintenance signals across the 575 neurons relative to previously described Error, Gain and Loss signals [2]. Each of the 3 signals reported in this paper was placed in a contingency matrix based on the counts of previously described neurons conveying the other signals. This contingency matrix was 4 (Error, Gain, Loss, or Neither) by 2 (with or without the signal). The bottom row shows the test statistics for homogeneity based on a chi-square test.

Conflict neurons multiplexed error, gain, and loss signals just in proportion to their incidence of sampling. In contrast, Event timing neurons were significantly less likely than chance to signal Loss and more likely to signal Error and Gain. Goal Maintenance neurons were significantly more likely than chance to multiplex with the Loss signal.

Supplementary Table 8 | Multiple linear regression results for relationship between ERPs and spiking.

a

ERP	Neuronal Type	Predictor	df	β	SE	*t*	p
N2	Conflict	Intercept	117	~0	0.023	-0.003	0.997
		spkUPPER		-1.04	0.290	-3.6	< 0.001
		spkLOWER		0.0135	0.296	0.046	0.963
N2	Event Timing	Intercept	97	~0	0.028	-0.003	0.998
		spkUPPER		-1.56	0.339	-4.60	< 0.001
		spkLOWER		0.547	0.327	1.67	0.097
N2	Goal Maintenance	Intercept	57	~0	0.036	0.0022	0.998
		spkUPPER		-0.652	0.510	-1.27	0.206
		spkLOWER		0.377	0.725	0.521	0.605
P3	Event Timing	Intercept	117	~0	0.024	0.0038	0.997
		spkUPPER		-0.157	0.356	0.441	0.660
		spkLOWER		-0.170	0.346	-0.492	0.623
P3	Event Timing	Intercept	97	~0	0.031	0.0005	0.999
		spkUPPER		-0.521	0.437	-1.193	0.236
		spkLOWER		-0.342	0.441	-0.775	0.440
P3	Goal Maintenance	Intercept	57	~0	0.029	0.011	0.991
		spkUPPER		2.09	0.383	5.46	< 0.001
		spkLOWER		0.61	0.422	1.47	0.148

b

ERP	Neuronal Type	Predictor	df	β	SE	*t*	p
N2	Conflict	Intercept	117	~0	0.024	-0.011	0.991
		spkUPPER		-0.84	0.319	-2.63	0.0098
		spkLOWER		-0.299	0.328	-0.911	0.364
N2	Event Timing	Intercept	97	~0	0.029	-0.015	0.988
		spkUPPER		-1.11	0.310	-3.57	< 0.001
		spkLOWER		0.502	0.349	1.44	0.153
N2	Goal Maintenance	Intercept	57	~0	0.036	0.0029	0.998
		spkUPPER		-0.397	0.527	-0.754	0.454
		spkLOWER		0.631	0.781	0.807	0.423
P3	Event Timing	Intercept	117	~0	0.024	0.0026	0.998
		spkUPPER		-0.129	0.337	0.382	0.703
		spkLOWER		-0.216	0.376	-0.575	0.566
P3	Event Timing	Intercept	97	~0	0.031	-0.0018	0.999
		spkUPPER		-0.460	0.422	-1.09	0.279
		spkLOWER		-0.621	0.484	-1.28	0.202
P3	Goal Maintenance	Intercept	57	~0	0.027	0.020	0.984
		spkUPPER		2.35	0.377	6.26	< 0.001
		spkLOWER		0.628	0.404	1.55	0.126

a, Results based on canceled trials with scatterplots in Figs 6f-g and Supplementary Figure 8a & 8c. **b,** Results based on activity difference between canceled and latency-matched no-stop-signal trials with scatterplots in Supplementary Fig 8b,d. In each model ERP voltage was the response variable and spike rate in the upper layers L2/3 (spkUPPER) and lower layers L5/6 (spkLOWER) was the predictor. No consideration for multiple comparison is needed in these statistics.

Supplementary Table 9 | Comparisons of modulation times between neurons in SEF, caudate, and VTA.

	Conflict (90.4 ± 8.5 ms, n = 75)	Event Timing (69.8 ± 6.3 ms, n = 84)	Goal Maintenance (117.7 ± 11.3 ms, n = 54)
**SNpc** (32.5 ± 8.1 ms, n = 25)	p < 0.001 *	p = 0.063	p < 0.001*
**SNpc + mesocortical latency** (133.1 ± 8.1 ms, n = 25)	p = 0.012*	p < 0.001 *	p = 0.578
**Caudate (Facilitation)** (114.4 ± 15.8 ms, n = 40)	p > 0.999	p = 0.123	p > 0.999
**Caudate (Suppression)** (116.2 ± 11.3 ms, n = 61)	p = 0.807	p = 0.005*	p > 0.999

One-way ANOVA, Onset Latency | Neuron Class, F(6, 249.897) = 8.291, p < 0.001
Tukey post-hoc comparisons (* = significant at adjusted p < 0.05 level).

Mean ± SEM latencies for type of neurons are shown beneath the cell type labels. A one-way independent measures ANOVA showed significant differences in modulation latencies between the different neuron types in SEF and the SNpc and caudate neurons (F(6,249.897) = 8.291, p < 0.001). The table reports outcomes of Dunn-adjusted Bonferroni post-hoc comparisons with statistically significant differences marked by an asterisk. SNpc DA neurons modulated significantly earlier than conflict (p < 0.001) and goal maintenance (p < 0.001) neurons but were not different from event timing neurons (p = 0.063). However, the estimated arrival times of DA spikes in SEF were not different from the modulation times of goal maintenance (p = 0.578) neurons but were significantly later than the modulation of event timing (p < 0.001) or conflict (p = 0.012) neurons. Meanwhile, although suppression after SSRT in the caudate nucleus arose significantly later than the modulation of event timing neurons in SEF (suppression p = 0.005), other caudate activity occurred simultaneously with event timing (facilitation p = 0.123), conflict (facilitation p > 0.999; suppression p = 0.807) and goal maintenance (facilitation p > 0.999; suppression p > 0.999) neurons.

SUPPLEMENTARY REFERENCES

1. Godlove, D.C., Maier, A., Woodman, G.F. & Schall, J.D. Microcircuitry of agranular frontal cortex: Testing the generality of the canonical cortical microcircuit. *J Neurosci* **34**, 5355-5369 (2014).

2. Sajad, A., Godlove, D.C. & Schall, J.D. Cortical microcircuitry of performance monitoring. *Nat Neurosci* **22**, 265-274 (2019).

3. Rapan, L., *et al.* Multimodal 3D atlas of the macaque monkey motor and premotor cortex. *Neuroimage* **226**, 117574 (2021).

4. Lowe, K.A. & Schall, J.D. Functional categories of visuomotor neurons in macaque frontal eye field. *eNeuro* **5**, eNeuro.0131-0118.2018 (2018).

5. Ogasawara, T., Nejime, M., Takada, M. & Matsumoto, M. Primate nigrostriatal dopamine system regulates saccadic response inhibition. *Neuron* **100**, 1513-1526 e1514 (2018).

6. Hanes, D.P. & Schall, J.D. Countermanding saccades in macaque. *Vis Neurosci* **12**, 929-937 (1995).

7. Murthy, A., Ray, S., Shorter, S.M., Schall, J.D. & Thompson, K.G. Neural control of visual search by frontal eye field: effects of unexpected target displacement on visual selection and saccade preparation. *J Neurophysiol* **101**, 2485-2506 (2009).

8. Schmidt, R., Leventhal, D.K., Mallet, N., Chen, F. & Berke, J.D. Canceling actions involves a race between basal ganglia pathways. *Nat Neurosci* **16**, 1118-1124 (2013).

9. Daw, N.D., Courville, A.C. & Touretzky, D.S. Representation and timing in theories of the dopamine system. *Neural Comput* **18**, 1637-1677 (2006).

10. Gibbon, J. Scalar expectancy theory and Weber's law in animal timing. *Psychol Rev* **84**, 279 (1977).

11. Janssen, P. & Shadlen, M.N. A representation of the hazard rate of elapsed time in macaque area LIP. *Nat Neurosci* **8**, 234-241 (2005).

12. Nelson, M.J., Boucher, L., Logan, G.D., Palmeri, T.J. & Schall, J.D. Nonindependent and nonstationary response times in stopping and stepping saccade tasks. *Atten Percept Psychophys* **72**, 1913-1929 (2010).

13. Starkweather, C.K., Babayan, B.M., Uchida, N. & Gershman, S.J. Dopamine reward prediction errors reflect hidden-state inference across time. *Nat Neurosci* **20**, 581-589 (2017).

14. Emmons, E., *et al.* Temporal learning among prefrontal and striatal ensembles. *Cerebral Cortex Communications* **1**, tgaa058 (2020).

15. Raftery, A.E. Bayesian model selection in social research. *Sociological methodology*, 111-163 (1995).

16. Grace, A.A. & Bunney, B.S. Nigral dopamine neurons: Intracellular recording and identification with L-dopa injection and histofluorescence. *Science* **210**, 654-656 (1980).

17. Thierry, A.M., Deniau, J.M., Herve, D. & Chevalier, G. Electrophysiological evidence for non-dopaminergic mesocortical and mesolimbic neurons in the rat. *Brain Res* **201**, 210-214 (1980).

18. Deniau, J.M., Hammond, C., Riszk, A. & Feger, J. Electrophysiological properties of identified output neurons of the rat substantia nigra (pars compacta and pars reticulata): evidences for the existence of branched neurons. *Exp Brain Res* **32**, 409-422 (1978).

19. Guyenet, P.G. & Aghajanian, G.K. Antidromic identification of dopaminergic and other output neurons of the rat substantia nigra. *Brain Res* **150**, 69-84 (1978).
