# Supplementary material for: Functional architecture of executive control and associated event-related potentials in macaques
Source: Nat Commun. 2022 Oct 21;13:6270. doi: 10.1038/s41467-022-33942-1 (PMC9586948; doi:10.1038/s41467-022-33942-1)
Supplement: Supplementary file 3 — Reporting Summary [file 41467_2022_33942_MOESM3_ESM.pdf]

nature portfolio

Corresponding author(s): Jeffrey D Schall

Last updated by author(s): Sep 8, 2022

Reporting Summary

Nature Portfolio wishes to improve the reproducibility of the work that we publish. This form provides structure for consistency and transparency in reporting. For further information on Nature Portfolio policies, see our Editorial Policies and the Editorial Policy Checklist.

Statistics

For all statistical analyses, confirm that the following items are present in the figure legend, table legend, main text, or Methods section.

n/a	Confirmed	
☐	☒	The exact sample size (n) for each experimental group/condition, given as a discrete number and unit of measurement
☐	☒	A statement on whether measurements were taken from distinct samples or whether the same sample was measured repeatedly
☐	☒	The statistical test(s) used AND whether they are one- or two-sided<br>*Only common tests should be described solely by name; describe more complex techniques in the Methods section.*
☐	☒	A description of all covariates tested
☒	☐	A description of any assumptions or corrections, such as tests of normality and adjustment for multiple comparisons
☐	☒	A full description of the statistical parameters including central tendency (e.g. means) or other basic estimates (e.g. regression coefficient) AND variation (e.g. standard deviation) or associated estimates of uncertainty (e.g. confidence intervals)
☐	☒	For null hypothesis testing, the test statistic (e.g. F, t, r) with confidence intervals, effect sizes, degrees of freedom and P value noted<br>*Give P values as exact values whenever suitable.*
☐	☒	For Bayesian analysis, information on the choice of priors and Markov chain Monte Carlo settings
☐	☒	For hierarchical and complex designs, identification of the appropriate level for tests and full reporting of outcomes
☒	☐	Estimates of effect sizes (e.g. Cohen's d, Pearson's r), indicating how they were calculated

Our web collection on statistics for biologists contains articles on many of the points above.

Software and code

Policy information about availability of computer code

Data collection	Visual stimulus generation, event timing, and task control were done with TEMPO (Reflective Computing). Eye position was sampled at 1 kHz using an Eyelink 1000 infrared eye-tracking system (SR Research). Behavioral and neural data was streamed to a MAP multichannel data acquisition system (Plexon Inc.).
Data analysis	MATLAB versions R2016a, R2017a, R2021a,b (MathWorks) and R version 11 (R Foundation for S tatistical Computing).

For manuscripts utilizing custom algorithms or software that are central to the research but not yet described in published literature, software must be made available to editors and reviewers. We strongly encourage code deposition in a community repository (e.g. GitHub). See the Nature Portfolio guidelines for submitting code & software for further information.

Data

Policy information about availability of data

All manuscripts must include a data availability statement. This statement should provide the following information, where applicable:

- Accession codes, unique identifiers, or web links for publicly available datasets
- A description of any restrictions on data availability
- For clinical datasets or third party data, please ensure that the statement adheres to our policy

The processed data used to: (a) generate figures and tables, and (b) produce the analyses in this study will be openly available online through the Open Science Framework.

Human research participants

Policy information about studies involving human research participants and Sex and Gender in Research.

Reporting on sex and gender	N/A
Population characteristics	N/A
Recruitment	N/A
Ethics oversight	N/A

Note that full information on the approval of the study protocol must also be provided in the manuscript.

Field-specific reporting

Please select the one below that is the best fit for your research. If you are not sure, read the appropriate sections before making your selection.

☒ Life sciences ☐ Behavioural & social sciences ☐ Ecological, evolutionary & environmental sciences

For a reference copy of the document with all sections, see nature.com/documents/nr-reporting-summary-flat.pdf

Life sciences study design

All studies must disclose on these points even when the disclosure is negative.

Sample size	The properties of some neurons in SEF are sufficiently well understood that a power analysis can guide data sampling. The variance of neural discharge rates scale with the mean, so with alpha = 0.05 and beta = 0.2, the nature of the modulation patterns that must be measured for the various studies require from 26 (Cohen d = 0.8) to 64 neurons (Cohen d = 0.5). However, many signals in SEF are less well characterized and exhibit more diversity and complexity. While a power analysis cannot be done without knowledge of population characteristics (Dell et al. 2002), we implement less formal algorithms to specify when to terminate data collection for a particular study. Based on the experience of our laboratory, neuron sampling within a region of interest for a particular study stops when two criteria are satisfied: (1) new discoveries demonstrate reliability as assessed by statistical analysis of neural modulation in relation to particular events (alpha = 0.05, beta = 0.2, from 26 (Cohen d = 0.8) to 64 neurons (Cohen d = 0.5)) and (2) mean values of derived neural measures do not change with further sampling over 2-3 experimental sessions. The time and number of samples needed to reach this termination criterion depends on how commonly sampled are the neurons of interest. The rarest response property that we can study effectively is exhibited by ~10% of a population. Our statistically specified goal, therefore, is to obtain a meaningful sample of this 10% in more than one monkey. Typically, the maximum number of such rare neurons that can be sampled within a given region of interest is between 20 and 30 (out of 200-300 neurons total). A sample of 40-60 such neurons would require 2 monkeys. In all cases, common results must be obtained from at least 2 monkeys to justify a rigorous, reproducible publication (Roelfsema & Treue 2014).
Data exclusions	The exclusion criteria are described in the Methods section of the paper. Neurons modulated in other intervals or not at all were not analyzed for this report. Also, for the analysis of various functional signals, trials in which two or more distinct events coincided in time were removed unless the confounding factors could be appropriately accounted for.
Replication	Replications consisted of repeated measures of different cortical sites (2 samples at each of 2 sites in each of 2 monkeys) in the same animal as well as repeating the same experiment in two monkeys.
Randomization	The experimental task involved psuedo-randomization of task conditions to ensure appropriate performance.
Blinding	The numbers of investigators, monkeys, and locations of samples are too few to enable any useful blinding.

Reporting for specific materials, systems and methods

We require information from authors about some types of materials, experimental systems and methods used in many studies. Here, indicate whether each material, system or method listed is relevant to your study. If you are not sure if a list item applies to your research, read the appropriate section before selecting a response.

Materials & experimental systems

n/a	Involved in the study
☒	☐ Antibodies
☒	☐ Eukaryotic cell lines
☒	☐ Palaeontology and archaeology
☐	☒ Animals and other organisms
☒	☐ Clinical data
☒	☐ Dual use research of concern

Methods

n/a	Involved in the study
☒	☐ ChIP-seq
☒	☐ Flow cytometry
☐	☒ MRI-based neuroimaging

Animals and other research organisms

Policy information about studies involving animals; ARRIVE guidelines recommended for reporting animal research, and Sex and Gender in Research

Laboratory animals	Data from two macaque monkeys: one male (M. radiata 8.8 kg, ~6 years old) and one female (M. mulatta 6 kg, ~8 years old)
Wild animals	No wild animals were used.
Reporting on sex	This study collected data from two macaque monkeys: one male and one female. Behavioral performance was equivalent across monkeys, and sex differences were not directly considered
Field-collected samples	No field-collected samples were used.
Ethics oversight	All procedures were in accordance with the National Institutes of Health Guidelines, the American Association for Laboratory Animal Care Guide for the Care and Use of Laboratory Animals and approved by the Vanderbilt Institutional Animal Care and Use Committee in accordance with the United States Department of Agriculture and Public Health Service policies.

Note that full information on the approval of the study protocol must also be provided in the manuscript.

Magnetic resonance imaging

Experimental design

Design type	MRIs were acquired to aid in placement of recording chambers and verifying angle of penetrations.
Design specifications	N/A
Behavioral performance measures	N/A

Acquisition

Imaging type(s)	Structural
Field strength	3T
Sequence & imaging parameters	3D turbo field echo anatomical sequence (TR= 8.729 ms; 130 slices, 0.70 mm thickness)
Area of acquisition	Whole brain scan

Diffusion MRI ☐ Used ☒ Not used

Preprocessing

Preprocessing software	N/A
Normalization	N/A
Normalization template	N/A
Noise and artifact removal	N/A
Volume censoring	N/A

Statistical modeling & inference

Model type and settings	N/A

Effect(s) tested	N/A

Specify type of analysis: [x] Whole brain [ ] ROI-based [ ] Both

Statistic type for inference (See Eklund et al. 2016)	N/A
Correction	N/A

Models & analysis

n/a	Involved in the study
[x]	[ ] Functional and/or effective connectivity
[x]	[ ] Graph analysis
[x]	[ ] Multivariate modeling or predictive analysis
